# Multiple Cationic Amphiphiles Induce a Niemann-Pick C Phenotype and Inhibit Ebola Virus Entry and Infection

**DOI:** 10.1371/journal.pone.0056265

**Published:** 2013-02-18

**Authors:** Charles J. Shoemaker, Kathryn L. Schornberg, Sue E. Delos, Corinne Scully, Hassan Pajouhesh, Gene G. Olinger, Lisa M. Johansen, Judith M. White

**Affiliations:** 1 Departmentof Cell Biology, University of Virginia, Charlottesville, Virginia, United States of America; 2 U.S. Army Medical Research Institute of Infectious Diseases (USAMRIID), Fort Detrick, Maryland, United States of America; 3 Zalicus Inc., Cambridge, Massachusetts, United States of America; 4 Departmentof Microbiology, University of Virginia, Charlottesville, Virginia, United States of America; University of Illinois at Chicago, United States of America

## Abstract

Ebola virus (EBOV) is an enveloped RNA virus that causes hemorrhagic fever in humans and non-human primates. Infection requires internalization from the cell surface and trafficking to a late endocytic compartment, where viral fusion occurs, providing a conduit for the viral genome to enter the cytoplasm and initiate replication. In a concurrent study, we identified clomiphene as a potent inhibitor of EBOV entry. Here, we screened eleven inhibitors that target the same biosynthetic pathway as clomiphene. From this screen we identified six compounds, including U18666A, that block EBOV infection (IC_50_ 1.6 to 8.0 µM) at a late stage of entry. Intriguingly, all six are cationic amphiphiles that share additional chemical features. U18666A induces phenotypes, including cholesterol accumulation in endosomes, associated with defects in Niemann–Pick C1 protein (NPC1), a late endosomal and lysosomal protein required for EBOV entry. We tested and found that all six EBOV entry inhibitors from our screen induced cholesterol accumulation. We further showed that higher concentrations of cationic amphiphiles are required to inhibit EBOV entry into cells that overexpress NPC1 than parental cells, supporting the contention that they inhibit EBOV entry in an NPC1-dependent manner. A previously reported inhibitor, compound 3.47, inhibits EBOV entry by blocking binding of the EBOV glycoprotein to NPC1. None of the cationic amphiphiles tested had this effect. Hence, multiple cationic amphiphiles (including several FDA approved agents) inhibit EBOV entry in an NPC1-dependent fashion, but by a mechanism distinct from that of compound 3.47. Our findings suggest that there are minimally two ways of perturbing NPC1-dependent pathways that can block EBOV entry, increasing the attractiveness of NPC1 as an anti-filoviral therapeutic target.

## Introduction

Ebolaviruses are members of the family Filoviridae. Infections by these viruses can produce acute hemorrhagic fever in humans and non-human primates, with species dependent lethality ranging from ∼50 to 90% [1], [2]. However, there are currently no approved vaccines or anti-viral therapeutics with which to combat ebolavirus infections [1], [3]. The virions are enveloped and contain a non-segmented negative-sense RNA genome. Morphologically, ebolaviruses are filamentous with a uniform diameter of ∼80 nm and lengths ranging from several hundred nanometers to several micrometers [4], [5]. The matrix protein VP40, the most abundant viral protein, drives virion formation [6], [7]. The surrounding viral membrane is densely studded with a trimeric glycoprotein (GP) whose first function is to attach viral particles to the cell surface. The virions are then internalized into the cell by a macropinocytic-like process, [8]–[12] and trafficked to late endosomes and perhaps lysosomes, where the cysteine proteases, cathepsin B and cathepsin L, proteolytically prime GP to a 19 kDa fusogenic form [13]–[17]. Fusion results in entry of the nucleocapsid into the cytoplasm, leading to genome replication and production of new virions [18].

Several cellular proteins required for the function and maturation of late endosomes (LE) and lysosomes (Lys) have recently emerged as ebolavirus entry factors. These include subunits of the HOPS complex and NPC1 [19]–[21], a multi-membrane spanning protein found in the limiting membrane of late endosomes/lysosomes (LE/Lys). When NPC1 is absent or dysfunctional, cholesterol and other substances accumulate in LE/Lys [22], [23]. Interestingly, the ability of NPC1 to facilitate cholesterol egress from LE/Lys is not required for NPC1 to promote ebolavirus entry [19], [20]. Although NPC1 can bind primed GP [24], its exact role(s) in ebolavirus entry has yet to be elucidated [25]. Nonetheless, NPC1 appears to be a good target for anti-filovirus intervention [19], [20]. For example, a novel inhibitor, compound 3.47, blocks binding of cathepsin-primed GP from Zaire ebolavirus (EBOV) to NPC1, and therefore blocks EBOV entry and infection [20].

The goal of this study was to identify additional small molecule EBOV entry inhibitors, and to probe their mechanisms of action. As a result, we identified six structurally related cationic amphiphiles that specifically block a late stage of EBOV entry. All of the inhibitors induced cholesterol accumulation in LE/Lys and those tested showed shifted dose-response curves in NPC1-overexpressing cells. However, none blocked the interaction of primed GP with NPC1. These results suggest that there are at least two ways of interfering with NPC1-dependent mechanisms that block EBOV entry into the cytoplasm, and that structurally-related cationic amphiphiles may prove clinically useful in combating EBOV infection.

## Materials and Methods

### Cells and Plasmids

HEK 293T cells (ATCC: CRL-11268) were maintained in high glucose Dulbecco’s Modified Eagle Medium (DMEM, Gibco Invitrogen) supplemented with 10% supplemented calf serum (Hyclone), 1% antibiotic/antimycotic, 1% L-Glutamine, and 1% Sodium Pyruvate. SNB19 human glioblastoma cells (ATCC: CRL-2219) were maintained in DMEM supplemented with 10% Fetal Bovine Serum (FBS, Gibco Invitrogen), 1% antibiotic/antimycotic, 1% L-Glutamine, and 1% Sodium Pyruvate. Vero E6 cells (ATCC: CRL-1586) were maintained in Eagle’s Minimum Essential medium (Gibco Invitrogen) supplemented with 10% FBS. JP17 parental Chinese Hamster Ovary cells (CHO) and JP17 cells overexpressing human NPC1 with a FLAG tag (CHO NPC1) were a gift of Frances Sharom and were maintained as previously described [23]. mCherry-VP40 was generated by sub-cloning the VP40 gene from pCAGGS VP40 (gift of Yoshihiro Kawaoka), and inserting it, in-frame, to the C-terminus of mCherry in the pmCherry-C1 vector (Clontech). β-lactamase VP40 was the gift of Lijun Rong.

### Chemical Reagents

Chemicals were obtained from the following sources: 5-(N-Ethyl-N-isopropyl) amiloride (EIPA; CAS 1154-25-2), clomiphene citrate (CAS 50-41-9), triparanol (CAS 78-41-1), BM 15766 (CAS 86621-94-5), SR 12813 (CAS 126411-39-0), and Filipin (CAS 480-49-9) (Sigma-Aldrich); bafilomycin A1 (CAS 88899-55-2) (LC Laboratories); U18666A (CAS 3039-71-2) and E64d (CAS 88321-09-9) (EMD Biosciences; Ro 48-8071 (CAS 161582-11-2) (BIOMOL); AY-9944 (CAS 366-93-8) (TOCRIS); alendronate sodium (CAS 129318-43-0) (ABATRA); terconazole (CAS 67915-31-5) (LEIRAS); amorolfine hydrochloride (CAS 106614-68-0) (LKT); colestolone (CAS 50673-97-7)(Fisher Scientific). Compound 3.47 was synthesized as previously described [20].

### Virus-like Particle (VLP) Production

Using Polyethylenimine (PolySciences Inc), 293T cells were co-transfected with plasmids encoding EBOV GP deleted for the mucin domain (GPΔ) along with three forms of the EBOV matrix protein: untagged VP40, VP40 tagged with β-lactamase, and VP40 tagged with mCherry [7], [26], [27]. The plasmids were transfected at a ratio of 6∶4∶9∶9 respectively. The mucin domain of GP has been shown to be dispensible for infection in tissue culture studies [28]. Control VLPs were prepared by replacing the plasmid encoding EBOV GPΔ with plasmids encoding VSV-G (gift of Michael Whitt) or LCMV GP (gift of Jack Nunberg). Media were harvested at 24 and 48 hr post-transfection, and cleared of debris twice by centrifugation at 1,500×g for 10 min at 4°C. VLPs were then pelleted through 20% sucrose in virus resuspension buffer (VRB; 130 mM NaCl, 20 mM Hepes, pH 7.4) by centrifugation for 2 hr at 112,398×g (25,000 rpm) in an SW28 rotor at 4°C. VLPs were resuspended overnight in 10% sucrose-VRB at 4°C, and then frozen at −80°C for long-term storage. VLPs were examined by immunofluorescence microscopy, which showed a high incorporation of GP into mCherry labeled VLPs. Analysis by negative stain electron microscopy showed that the VLPs were densely studded with GP spikes [29].

### VLP Internalization and Cytoplasmic Entry Assays

The day before each experiment, 100,000 SNB19 cells were seeded into each well of a 48-well plate. All internalization and cytoplasmic entry assays were conducted in serum-free Optimem I media (Gibco). For inhibitor studies, SNB19 cells were pretreated with either DMSO (mock) or the indicated concentration of inhibitor for 1 hr at 37°C, and inhibitors were maintained in all following steps. Cells were then pre-chilled to 4°C for 15 min, and VLPs were bound to cells by spinfection at 250×g for 1 hr at 4°C. Following 2 washes with inhibitor (where appropriate), cells were warmed to 37°C for 1 hr or 3 hr in a 5% CO_2_ incubator for the internalization and cytoplasmic entry assays, respectively. Cells were then processed as indicated below.

For the internalization assay, samples were treated with 0.5% Trypsin-EDTA (Gibco) for 30 min at 4°C to strip surface associated particles. Cells were then lifted by gentle pipeting, washed, fixed, and analyzed on an LSR Fortessa flow cytometer (Becton Dickinson). Gating in the mCherry channel was determined from samples that were not exposed to VLPs, and data are presented as percent of cells with mCherry fluorescent signal. Cells exposed to VLPs, but maintained at 4°C throughout the experiment, served as a control confirming the efficiency of protease stripping of non- internalized VLPs. For the cytoplasmic entry assay, samples were washed once with loading buffer (phenol red free DMEM supplemented with 2 mM L-glutamine, 2.5 µM probenecid, 25 mM Hepes, and 200 nM Bafilomycin) post-incubation. Cells were then incubated in the dark for 1 hr at RT in loading buffer supplemented with 1 µM CCF2-AM (Invitrogen), a β-lactamase substrate. Cells were washed with loading buffer and then incubated overnight in the dark at RT with 10% FBS-loading buffer. Cells were lifted with trypsin, fixed, and analyzed on a FACSCaliber flow cytometer. Cytoplasmic entry was assessed by the degree of positive shift by cells in the blue channel (447 nm emission) relative to cells that did not receive any VLPs. All flow cytometric data were analyzed with FlowJo software.

We validated the multi-purpose EBOV VLPs using chemical inhibitors and mutant GP proteins. For example, we showed that the cathepsin inhibitor, E64d, as well as the F535R fusion loop mutation in GP, block EBOV-GP-VLP entry into the cytoplasm while having no effect on VLP internalization. We further characterized the kinetics of VLP internalization and cytoplasmic entry and, interestingly, found that cathepsin priming to 19 kDa GP is not a rate-limiting step for cytoplasmic entry [29].

### Live Virus Infections

Infections were conducted with EBOV engineered to express the green fluorescent protein (EboZ-eGFP) [30]. Briefly, 40,000 Vero E6 cells were seeded in 96 well plates and allowed to grow overnight at 37°C, 5% CO_2_. The next day, drugs were added to the cells at the indicated concentrations, followed by virus at a multiplicity of infection of approximately 0.01. Cells were then incubated for 48 hrs at 37°C, 5% CO_2_ and GFP fluorescence was read with a spectrofluorometer. All infections were performed in bio-safety level 4 (BSL-4) facilities at USAMRIID: personnel wear positive-pressure protective suits (ILC Dover, Frederica, DE) fitted with HEPA filters and umbilical-fed air. USAMRIID is registered with the Centers for Disease Control (CDC) Select Agent Program for the possession and use of biological select agents and toxins and has implemented a biological surety program in accordance with U.S. Army regulation AR 50-1 “Biological Surety”. All procedures were conducted as previously described [31].

Cell viability (in response to drugs) was assessed in uninfected sister wells that had been exposed to inhibitors, and was determined using the Promega Cell Titer-Glo luminescent cell viability kit.

### Pseudovirion Infection

GFP encoding VSV-GPΔ was produced as described previously [14]. The day before each experiment, 20,000 parental CHO or NPC1 overexpressing CHO NPC1 cells were seeded in each well of a 96 well micro-titer plate. On the day of the experiment, cells were pre-treated with the indicated concentration of inhibitor for 1 hr at 37°C in a 5% CO_2_ incubator. Cells were then infected in the presence or absence of inhibitor with VSV-GPΔ at a multiplicity of infection of ∼0.5. Infections were allowed to proceed for 18 hr at which time samples were fixed and analyzed by flow cytometry for GFP expression [14].

### NPC1-19 kDa GP Co-IP

A fraction enriched for LE/Lys was prepared from CHO NPC1 cells essentially as described [20], except that the cells were first sheared with a cell cracker [32] with a cylinder bore of 0.25 inches and an 0.2496 inch diameter ball bearing. Briefly, CHO NPC1 cells were lifted and resuspended at a density of 1.5×10^6^ to 2.5×10^6^ cells/mL in HMB buffer (250 mM sucrose, 1 mM EDTA, 10 mM HEPES, pH 7.0) with protease inhibitors. After allowing cells to swell for 10 min on ice, cells were passed through the cell cracker 7 times. The cell homogenate was examined by microscope to confirm that plasma membranes (but not nuclear membranes) had been disrupted. Nuclei were then pelleted, and the post-nuclear supernatant was then centrifuged at 15,000×g (9,400 rpm) in an SW41 rotor for 32 minutes at 4°C. Membrane pellets were then resuspended in HMB buffer, and the protein concentration was determined by BCA and adjusted to ∼2 mg/mL. After disrupting the membranes with 20 mM methionine methyl-ester for 1 hr at RT, ∼150 µg of disrupted NPC1-enriched membranes were pre-incubated with the indicated concentration of inhibitor for 30 min at RT. Next, 3 µg of soluble EBOV GP trimeric ectodomain (gift of Lianying Yan and Chris Broder), either full length (negative control) or cleaved to the 19 kDa form were added. Cleavage to 19 kDa GP was accomplished by treating the full length ectodomain (0.5 mg/mL in 20 mM Mes, 20 mM Hepes, 130 mM NaCl) with 0.2 mg/mL of thermolysin (containing 2 mM Ca^+2^) for 1 hr at 37°C [17]. Reactions were quenched with 500 µM phosphoramidon and the GP proteins were frozen at −80°C until use. Cleavage of GP to 19 kDa was confirmed by western blot (data not shown). After incubating with ectodomain proteins for 1 hr at RT, the membranes were disrupted with 10 mM CHAPSO (EMD Biosciences) in TNE buffer (10 mM Tris, 150 mM NaCl, 1 mM EDTA, pH 7.4). After clearing debris at 21,100×g (14,800 rpm) for 10 min at 4°C, supernatants were incubated with anti-FLAG coated magnetic beads (Sigma) overnight at 4°C with end-over-end rotation. The magnetic beads were then collected, washed 2 times with bead wash buffer (10 mM CHAPSO in TNE (pH 7.4) w/protease inhibitors), and exposed to 0.1M glycine, pH 3.0 for 5 min at RT to elute bound GP and NPC1-FLAG from the anti-FLAG beads. The eluted proteins were then denatured in SDS sample buffer with DTT at a final conc. of 9 mM, and run on an Any KD® SDS-PAGE gel (Biorad). Proteins were transferred to nitrocellulose, and blotted with polyclonal antibodies against EBOV GP (gift of Paul Bates) and NPC1 (ThermoFischer Scientific: PA1-16817). Protein blots were imaged on an Odyssey® Infrared Imaging System (LI-COR Biosciences), and band intensities were quantified using Odyssey application software (version 3.0.16**)** and reported as the band intensity of GP or GP_19 kDa_ divided by the band intensity of NPC1-FLAG.

### Cholesterol Accumulation Assay

Cholesterol accumulation was monitored by staining SNB19 cells with filipin, as described in Kobayashi, et al. [33]. The day before an experiment, 50,000 cells were plated on glass coverslips in a 24 well plate. The next day, cells were treated with inhibitors at the indicated concentrations for 21 hr. After fixation with 4% paraformaldehyde, cells were washed twice with PBS, incubated in 50 µg/mL filipin (Sigma-Aldrich) in PBS for 1 hr at RT, and washed 3 times with PBS, after which the coverslips were mounted and imaged on a Zeiss Axio Observer fluorescence microscope. Samples were scored for cholesterol accumulation in LE/Lys by a blind observer in a minimum of 3 separate experiments. Representative images were inverted for clarity, and are shown with uniform adjustments to brightness and contrast across all images.

### Endosomal Acidification and Cathepsin Activity Assays

Effects of inhibitors on endosomal pH and cathepsin B and L activity in SNB19 cells were assessed as described in [34]. Briefly, after a 1 hr incubation at 37°C with inhibitor at the indicated concentration, cathepsin B activity in cell lysates was determined using the cathepsin B substrate Z-Arg-Arg-7-AMC (Calbiochem). Cathepsin L was assayed using the cathepsin L substrate Z-Phe-Arg-7-AMC (Calbiochem) in the presence of 1 µM CA074, a cathepsin B inhibitor (Calbiochem). Combined activity of cathepsins B and L was assayed as above using the cathepsin L substrate without any inhibitor. Fluorescence was measured using a fluorometer.

Endosomal acidification was assessed using Lysotracker Red (Molecular Probes) as a probe for low pH organelles. Cells were pre-treated with or without inhibitor for 1 hr at 37°C, 5% CO_2_ and then incubated with 50 nM Lysotracker Red for an additional 30 min (with inhibitor where appropriate). After this, cells were fixed and analyzed by fluorescence microscopy. Changes made to image contrast and brightness were made uniformly across all images.

### Analyses of Chemical Structures

The p*K*_a_ values were determined using the ACD/p*K*_a_ program, which quickly and accurately predicts the acid–base ionization constant of a wide range of organic compounds. It uses Hammett equations derived from a library of highly curated compounds to predict an aqueous p*K*_a_ value. In addition, two reference databases are available that offer quick look-ups of published data: one contains >31,000 experimental p*K*_a_ values for approximately 16,000 compounds in aqueous solutions; the other provides experimental data for more than 2,000 molecules in non-aqueous solvents. This software is used by the majority of pharmaceutical companies worldwide, and has been tested on a wide variety of chemical classes [35]–[37]. The clogP is the log of the octanol-water partition coeffient, P, and is related to the hydrophobic character of the molecule. It is useful in predicting solubility, drug-likeness, and permeability. The values were determined using the ChemDraw program, which calculates the octanol-water coefficient for a wide range of neutral compounds under standard conditions, at 25°C. The calculations are provided with 95% confidence intervals [38], [39].

## Results and Discussion

### A Subset of Sterol Synthesis Inhibitors Block EBOV Entry and Infection

**Figure 1:**
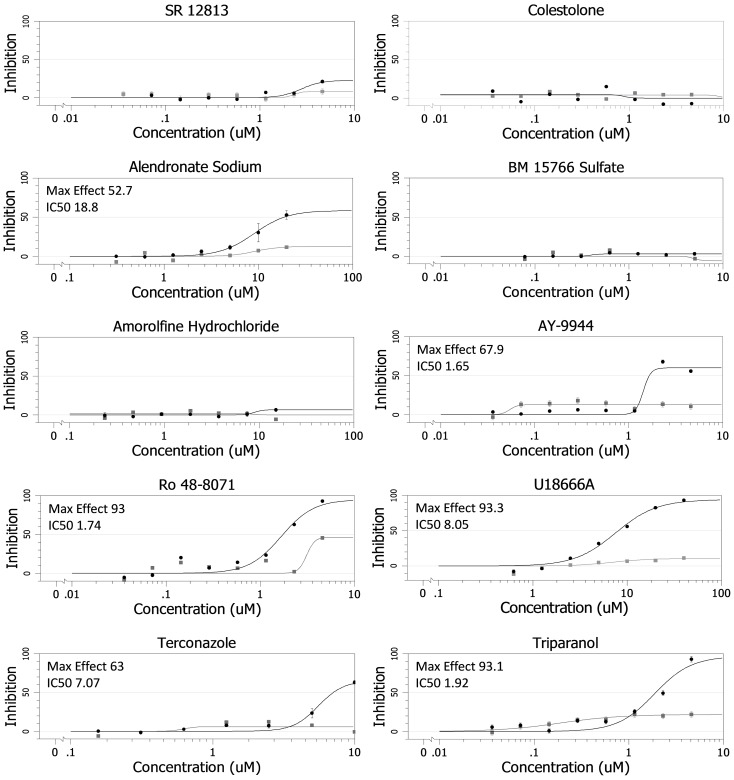
Effects of Sterol Pathway Inhibitors on EBOV Infection. Dose response curves for the indicated sterol pathway inhibitors are shown. The compounds were evaluated, in parallel at the indicated concentrations, for their ability to inhibit EBOV infection (black) and for inhibition of cell proliferation (gray) in Vero cells. The maximal % inhibition and the IC_50_ (µM) for their effects on EBOV infection are indicated. Data for clomiphene are presented in Johansen, et al. (manuscript in preparation).

We recently found that clomiphene, an FDA-approved drug, blocks EBOV infection of Vero cells (IC_50_ = 2.42 µM). Clomiphene also inhibits EBOV in a mouse model of infection, providing 90% survival at day 28 post-infection. Although best known as an estrogen receptor antagonist, clomiphene inhibits EBOV infection whether or not target cells express estrogen receptors (manuscript in preparation). Clomiphene is also known as an inhibitor of squalene epoxidase, a key enzyme in the pathway of sterol synthesis [40], a pathway necessary for infection by several viruses including hepatitis C virus (HCV) [41]. We therefore tested eleven sterol synthesis pathway inhibitors, previously shown to affect replication of HCV, for their effects on EBOV infection [40]. Seven of the eleven compounds tested inhibited infection by replication competent EBOV; the other four had no significant effect (Fig. 1). Since some, but not all, of the sterol synthesis inhibitors blocked EBOV infection, it appears that, unlike for HCV [40], inhibition of sterol synthesis *per se* is not the mechanism by which these drugs block EBOV infection.

**Figure 2:**
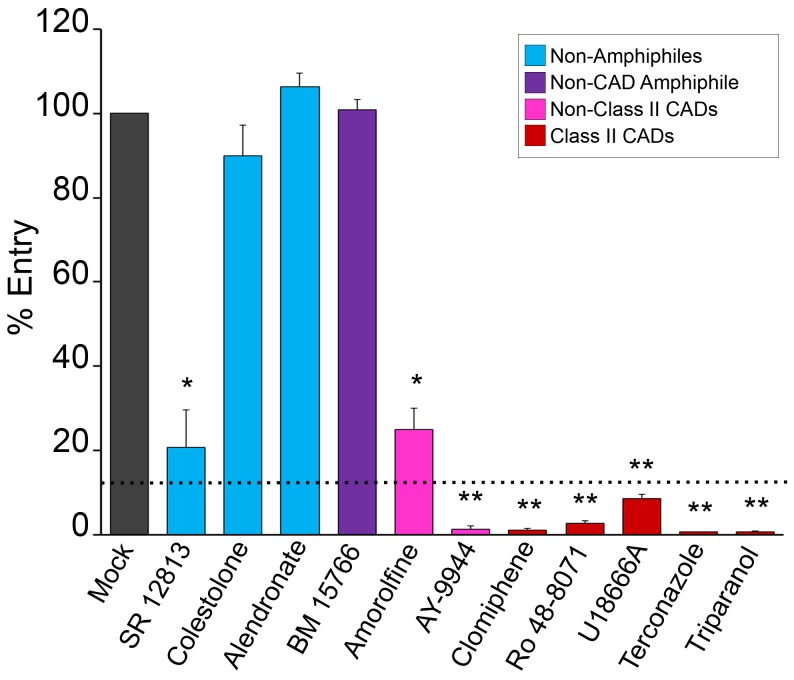
Effects of sterol pathway inhibitors on EBOV VLP entry. SNB19 cells were pretreated with inhibitor for 1 hr, and EBOV VLP-GPΔ was then bound by spinfection at 4°C for 1 hr. Cells were then washed in media with inhibitor, incubated at 37°C for 3 hr (in inhibitor), and then processed for VLP entry as described in the Materials and Methods section. Compounds were tested in multiple (n) experiments (each in triplicate) at either the highest concentration under which no toxicity was observed (for compounds that did not inhibit infection), or at the concentration that resulted in maximum inhibition of EBOV infection (Fig. 1) with minimal toxicity: SR 12813 (5 µM), (n = 4), colestolone (10 µM), (n = 5), alendronate (20 µM), (n = 3), BM 15766 (2 µM), (n = 4), amorolfine (6 µM), (n = 4), AY-9944 (5 µM), (n = 3), clomiphene (5 µM), (n = 9), Ro 48-8071 (5 µM, (n = 6), U18666A (5 µM), (n = 7), terconazole (10 µM), (n = 4), and triparanol (5 µM), (n = 3). Error bars represent standard error: * (P<2.96×10^−3^) or ** (P<6.14×10^−5^). Dashed line represents the observed threshold for entry inhibition needed to observe corresponding inhibition of live EBOV infection (Fig. 1). As indicated in the key, colors denote classes of molecules.

We next tested the panel of eleven compounds for their effects on EBOV GPΔ-mediated entry into the cytoplasm of host cells. We did this using VLPs containing EBOV VP40 fused with β-lactamase [27], [42] as well as EBOV GPΔ on their surface [29]. When the VLPs fuse with the limiting membrane of a LE/Lys, VP40 β-lactamase reaches the cytoplasm, where it can cleave a loaded substrate, causing a fluorescent color shift that can be read by flow cytometry [43]. As seen in Fig. 2, eight of the compounds inhibited VLP entry. Six of them (clomiphene, Ro 48-8071, U18666A, terconazole, AY- 9944, and triparanol) inhibited entry >91% at the concentration tested (dashed line in Fig. 2), an apparent threshold (in this single cycle assay) for a corresponding inhibition of multiple cycles of replication with authentic virus (Fig. 1).

### Newly-identified Strong EBOV Entry Inhibitors are Cationic Amphiphilic Drugs

Structures for the eleven compounds analyzed in this study are given in Supp. Fig. 1. Among them, eight are amphiphiles and three are not (Table 1). Among the three non-amphiphiles (blue in Fig. 2), two (SR12813 and colestolone) had no effect on EBOV infection (Fig. 1). The third, alendronate, inhibited infection (Fig. 1), but did not inhibit entry (Fig. 2), indicating that it blocks a post-entry step in the EBOV life cycle.

**Table 1:** Summary: Properties and effects of sterol synthesis pathway inhibitors on EBOV entry and infection and on cholesterol accumulation in LE/Lys.

Compound	TargetEnzyme^a^	Inhibit EBOVInfection (IC_50_)^b^	Inhibit VLPEntry^c^(% +/− SD)	IncreaseCHOLin LE/Lys^d^	ChemicalStructure	MW	p*K*_a_ g	clogP^g^
SR 12813	HMGCR	No	79.3+/−8.9	No	Not amphiphile	504.5	9.5	6.6
Colestolone	HMGCR	No	10.0+/−7.3	No	Not amphiphile	400.6	15.1	7.2
Alendronate	FPPS	Yes (18.8 µM)	−6.3+/−3.3	No	Not amphiphile	248.1	14.4	−5.6
BM 15766	d7R	No	−.7+/−2.5	No	Zwitterionic amphiphile	384.0	8.4	1.9
Amorolfine	c14d8	No	75.0+/−5.0	No	CAD^f^	317.0	7.1	6.4
**AY 9944**	**d7/d14R**	**Yes (1.65 µM)**	**98.7+/−0.8**	**Yes**	**CAD**	**391.4**	**9.1**	**6.4**
**Clomiphene**	**SQLE**	**Yes (2.42 µM)**	**98.8+/−.4**	**Yes**	**Class II CAD**	**405.9**	**9.6**	**7.2**
**Ro 48-8071**	**OSC**	**Yes (1.74 µM)**	**97.2+/−.6**	**Yes** e	**Class II CAD**	**434.3**	**8.8**	**5.7**
**U18666A**	**OSC**	**Yes (8.00 µM)**	**91.4+/−1.1**	**Yes**	**Class II CAD**	**387.6**	**9.7**	**5.1**
**Terconazole**	**c14dM**	**Yes (7.07 µM)**	**99.3+/−0**	**Yes**	**Class II CAD**	**532.5**	**8.8**	**4.8**
**Triparanol**	**d24R**	**Yes (1.92 µM)**	**99.3+/−0.3**	**Yes**	**Class II CAD**	**438.0**	**9.6**	**6.7**

a abbreviations of target enzymes in cholesterol synthesis pathway: HMGCR, HMG CoA Reductase; FPPS, Farnesyl pyrophosphate synthase; d7R, Sterol delta-7 reductase; c14d8, Lanosterol C14-demethylase/sterol delta; d7/d14R, Sterol delta-7 and delta-14 reductase; SQLE, Squalene epoxidase; OSC, 2,3 Oxidosqualene cyclase; c14dM, Lanosterol C14-demethylase; d24R, Sterol delta-24 reductase.

b data for clomiphene are from Johansen et al., in revision; all other data are from Fig. 1.

c data from Fig. 2.

d data from Fig. 5; CHOL, cholesterol.

e Ro 48-8071 appeared to induce less CHOL accumulation than the other inhibitors scored ‘yes’ for this phenotype.

f CAD, Cationic amphiphilic drug.

g p*K*_a_ and cLogP were calculated as described in the Methods section. All compounds highlighted in bold were shown to robustly block EBOV GP-mediated VLP entry (≥90% inhibition) and infection by authentic EBOV.

Among the eight amphiphiles, six strongly inhibited EBOV entry (≥91%) and infection. The six strong inhibitors are all cationic amphiphilic drugs (CADs), and five of the six are class II CADs (clomiphene, Ro 48-8071, U18666A, terconazole, and triparanol; red in Fig. 2); a class II CAD is an amphiphilic amine with clearly segregated hydrophobic and hydrophilic segments (Suppl. Fig. S1, Table 1). The other three amphiphiles tested were BM 15766 (a zwitterionic amphiphile), AY-9944 and amorolfine. The zwitterionic amphiphile, BM 15766 (purple in Fig. 2), did not inhibit EBOV GP-mediated entry or infection, suggesting the importance of positive molecular charge for this entry inhibition mechanism. Interestingly, among the two non-class II CADs (pink in Fig. 2), AY-9944 strongly inhibited EBOV GP-mediated entry and infection, while amorolfine had only a modest effect on entry and no effect on infection. The p*K*_a_ of AY-9944, an EBOV inhibitor, is 9.1, whereas that of amorolfine, a non-inhibitor, is 7.1. All of the other strong EBOV entry inhibitors identified in this screen have p*K*_a_ values >8.8. The relatively low p*K*_a_ of amorolfine might result in lower sequestration in LE/Lys [44], which could, in turn, account for its limited effect on EBOV entry, despite being a CAD.

Thus, the common features of the six strong EBOV entry inhibitors identified in this analysis (last six compounds in Fig. 2) are that they are all CADs (five of the six being class II CADs) that contain one or more secondary or tertiary amines protonatable at physiological pH (Suppl. Fig. S1; Table 1). Furthermore, all six strong CAD inhibitors have molecular weights in the range of 388 to 532, clogP values between 4.8 and 7.2, and p*K*_a_ values between 8.8 and 9.7. All six also cause cholesterol accumulation in endosomes (see below).

### CADs Inhibit a Late Stage of EBOV Entry

**Figure 3:**
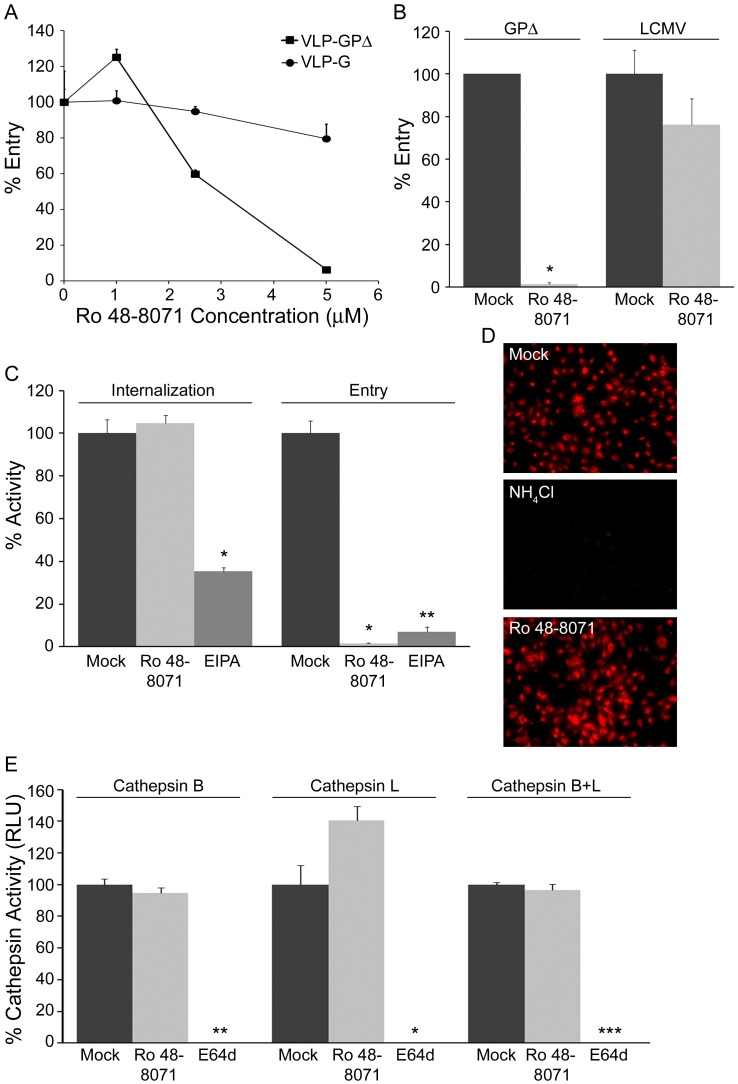
Ro 48-8071 inhibits EBOV entry at a post internalization step and does not inhibit endosome acidification or cathepsin levels. Effects of Ro 48-8071 (indicated concentration in A, 5 µM in B-E) on: (A) VLP-GPΔ and VLP-G entry; one representative of two experiments (done in triplicate). (B) VLP-GPΔ and VLP-LCMV entry; one representative of three experiments (done in duplicate). (C) VLP-GPΔ internalization and entry; 50 µM EIPA was used as the positive control for an inhibitor of EBOV internalization [8]; 10 µM EIPA was used as the control for the entry assay (50 µM EIPA caused high background fluorescence in the entry assay); one representative of two experiments (done in triplicate). (D) Low endosomal pH was detected by incubating cells with Lysotracker Red; 10 mM NH_4_Cl was used as the control for pH neutralization; representative images from multiple coverslips from a single experiment. (E) Cathepsin B, L, and combined B/L activity; 10 µM E64d was used as the positive control for inhibition of cysteine protease activity; results from a single experiment performed in duplicate. In all assays, SNB19 cells were pre-treated with the indicated concentration of inhibitor for 1 hr at 37°C, and inhibitors were maintained throughout the assays. Error bars represent standard deviation from the mean of mock-treated samples: * (P<.01), ** (P<.001), or *** (P<.0001).

**Figure 4:**
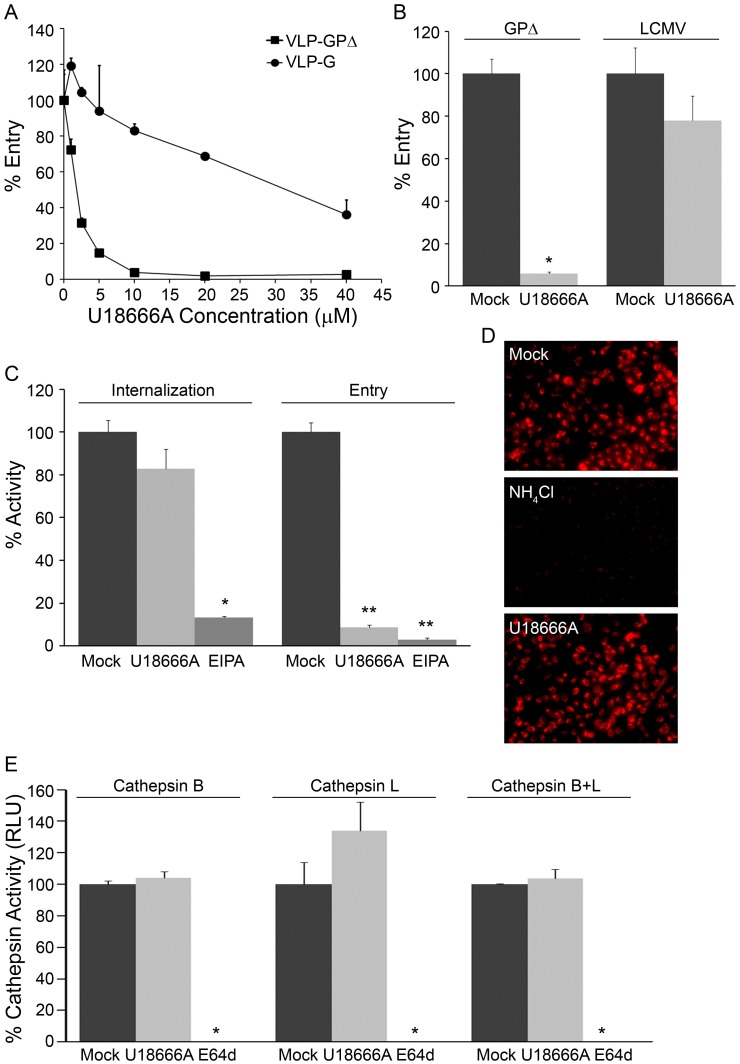
U18666A inhibits EBOV entry at a post internalization step and does not inhibit endosome acidification or cathepsin levels. Effects of U18666A (indicated concentration in A, 5 µM in B-E) on: (A) VLP-GPΔ and VLP-G entry; one representative of two experiments (done in triplicate). (B) VLP-GPΔ and VLP-LCMV entry; one representative of three experiments (done in duplicate). (C) VLP-GPΔ internalization and entry (controls as in Fig. 3C); one representative of two experiments (done in triplicate). (D) Endosomal pH detected by Lysotracker Red; 10 mM NH_4_Cl was used as the control for pH neutralization; representative images from multiple coverslips from a single experiment (E) Cathepsin B, L, and combined B/L activity; E64d was used as the positive control as in Fig. 3E; results from a single experiment performed in duplicate. In all assays, SNB19 cells were pre-treated with the indicated concentration of inhibitor for 1 hr at 37°C, and inhibitors were maintained throughout the assays. Error bars represent standard deviation from the mean of mock-treated samples: * (P<.01), ** (P<.001), or *** (P<.0001).

We characterized the mode of action of two of the CADs, Ro 48-8071 and U18666A, in more detail. As seen in Figs. 3A and 3B, Ro 48-8071 strongly inhibited entry of VLP-GPΔ into the cytoplasm, while having only small effects on the cytoplasmic entry of VLPs coated with either VSV G (Fig. 3A) or LCMV GP (Fig. 3B), indicating specificity for EBOV GP-mediated entry rather than a general impairment in function of early or late endosomes. Moreover, Ro 48-8071 did not inhibit VLP-GPΔ internalization from the cell surface (Fig. 3C), endosomal acidification (Fig. 3D), or cathepsin activity levels (Fig. 3E). U18666A (Fig. 4) and clomiphene (manuscript in preparation) behaved similarly to Ro 48-8071 in all of these respects. Hence, these CADs most likely impede EBOV entry by blocking events closely associated with EBOV fusion with the limiting membrane of a LE/Lys.

### CADs Inhibit EBOV Entry through an NPC1-dependent Pathway

**Figure 5:**
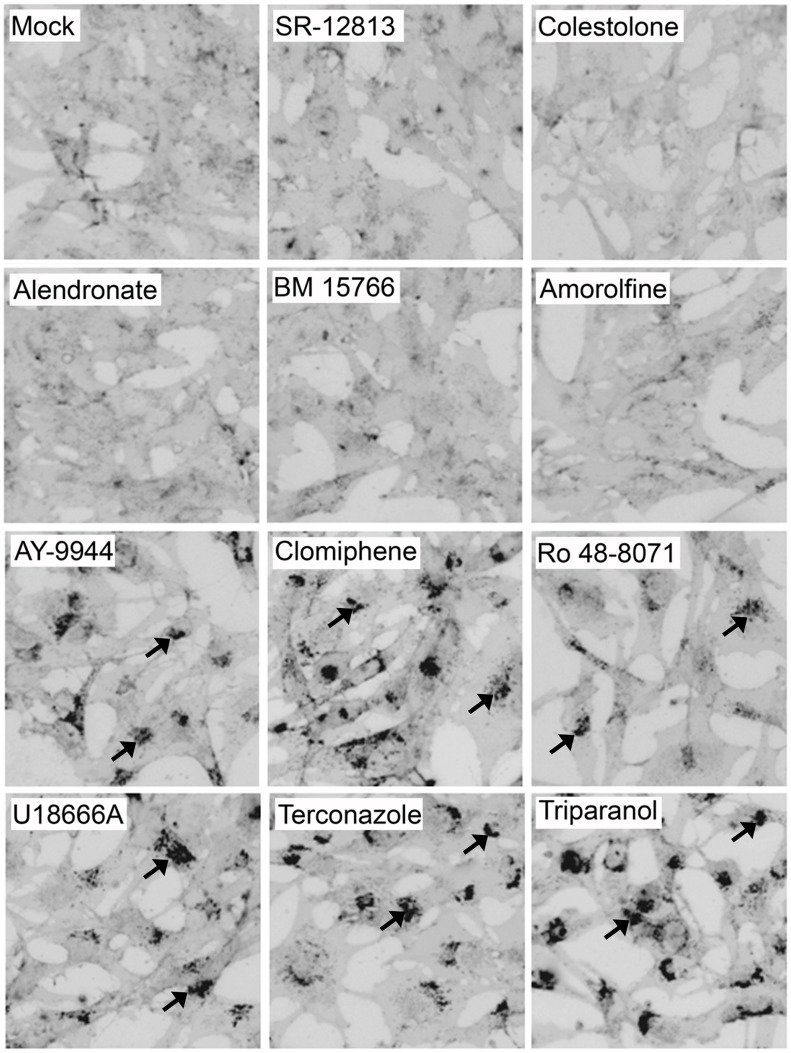
CADs that strongly inhibit EBOV entry and infection cause cholesterol accumulation in LE/Lys. SNB19 cells were treated for 21 hr with either DMSO or inhibitor (concentrations as in Fig. 2). Cells were then fixed, stained with filipin, and imaged on a fluorescence microscope. Images were inverted and uniformly adjusted for contrast and brightness. Representative images are shown. Arrows indicate sites of cholesterol accumulation. Each compound was tested at least 3 times, and scored (+/−) by a blind observer (Table 1).

U18666A, one of the inhibitors identified in our set, as well as by Carette, et al. [19], is known to induce many of the defects seen in NPC1-deficient cells, notably cholesterol accumulation in LE/Lys [33]. We therefore tested our panel of compounds for their effects on cholesterol accumulation [45]. As seen in Fig. 5, the six CADs that potently inhibited VLP-GPΔ entry (>91%) and EBOV infection all induced cholesterol accumulation in LE/Lys (bottom two rows, Fig. 5). In contrast, the compounds that did not strongly inhibit VLP-GPΔ entry did not cause detectable cholesterol accumulation (top two rows, Fig. 5). Two other CADs, which are not sterol synthesis pathway inhibitors, also block a late stage of EBOV GP-mediated entry, inhibit EBOV infection, and cause the cholesterol accumulation phenotype (unpublished data). Hence, among the CADs tested, there is a strict correlation between inhibition of EBOV entry and induction of cholesterol accumulation.

**Figure 6:**
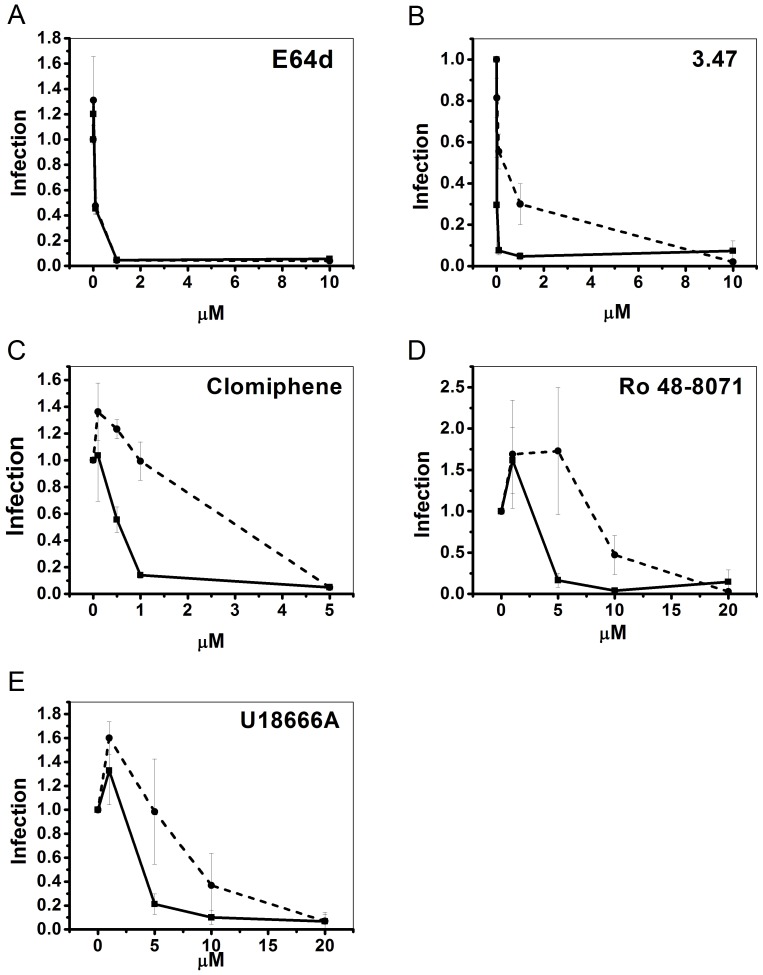
CADs inhibit EBOV GP-mediated infection in an NPC1-dependent manner. Parental CHO cells (−−−−) and stably overexpressing CHO NPC1 cells (− − −) were pre-treated with the indicated concentration of inhibitor for 1 hr at 37°C, and then infected with VSV-GPΔ for 18 hr in the continued presence of inhibitor. Each concentration of inhibitor was tested (in duplicate) in the following number of experiments: E64d (n = 2), compound 3.47 (n = 2), clomiphene (n = 3), Ro 48-8071 (n = 3), and U18666A (n = 4). Infection values were normalized to DMSO treated samples and averaged across experiments. Error bars represent standard error.

The results shown in Fig. 5 suggested that the six CADs that inhibit EBOV entry exert their effects through an NPC1-dependent pathway. To test this hypothesis, we compared the effects of three of them, clomiphene, Ro 48-8071, and U18666A, on EBOV GP-mediated pseudovirion infection in CHO cells expressing basal or heightened levels of NPC1. (The VLP cytoplasmic entry assay could not be performed in the NPC1 overexpressing cells due to beta lactamase activity from the ampicillin resistant plasmid used to create the cell line [46]). As a negative control, we tested the cysteine protease inhibitor E64d, which blocks EBOV entry by inhibiting cathepsins B and L (i.e. functions independent of NPC1). As predicted, E64d inhibited EBOV GP-mediated infection with the same dose-dependence in parental and NPC1 overexpressing cells (Figure 6A). As shown previously, higher concentrations of compound 3.47, a piperazine that inhibits EBOV infection by blocking GP binding to NPC1 [20], were required to inhibit EBOV GP-mediated infection in NPC1 overexpressing vs. parental cells (Figure 6B). Similar to compound 3.47, higher concentrations of each of the three CADs tested were required to inhibit EBOV GP-mediated infection of the NPC1-overexpressing cells relative to parental cells (Figs. 6C–E). Our findings for U18666A in the two cell lines (Fig. 6E) are consistent with the observation that higher concentrations of U18666A are required to induce cholesterol accumulation in cells that overexpress NPC1 [47]. Collectively, the results in Figs. 5 and 6 support our proposal that the CADs that block EBOV entry do so through an NPC1-dependent pathway. They do not, however, alter cellular expression levels of NPC1 (Supplemental Fig. S2).

The inhibitory effect of the CADs on EBOV entry is not likely a direct consequence of their induction of detectable cholesterol accumulation. Our reasoning is that clomiphene, Ro 48-8071, and U18666A all inhibit EBOV entry well before they cause detectable cholesterol accumulation. For the entry experiments, cells are pretreated with inhibitors for 1 hr and then maintained in inhibitor during a 3 hr entry period. Furthermore, the CADs can be added at the initiation of VLP-GPΔ internalization and still strongly block entry (unpublished data), whereas a minimum of ∼8 hrs is required to see detectable cholesterol accumulation [48]. Since EBOV GP-mediated entry begins within 1 hr following binding [29], the CADs appear to act rapidly on a cellular target that is critical for EBOV entry.

### CADs do not Disrupt the *in vitro* Interaction of Primed EBOV GP with NPC1

**Figure 7:**
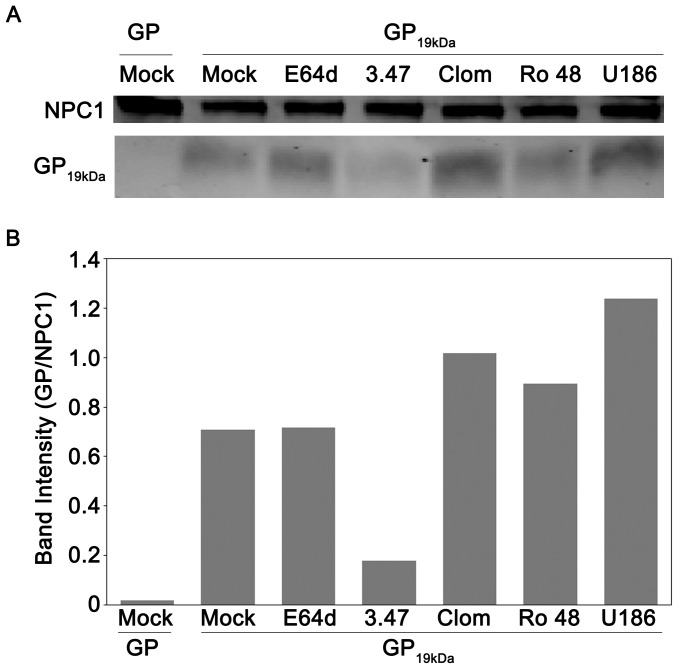
CADs do not disrupt the interaction of 19 kDa GP and NPC1. (A) NPC1-FLAG-enriched LE/Lys membranes from CHO NPC1 cells were disrupted and then incubated with inhibitors for 30 min at RT: mock (4% DMSO), E64d (10 µM), compound 3.47 (13 µM), clomiphene (242 µM), Ro 48-8071 (174 µM), and U18666A (800 µM); each inhibitor was used at a concentration 100 fold over its IC_50_ for inhibition of infection. The samples were then incubated with 3 µg uncleaved (GP) or cleaved (GP_19 kDa_) EBOV GP ectodomains for 1 hr at RT. Samples were then lysed, and incubated overnight with anti-FLAG beads. Bound NPC1 and GP were then eluted from beads, and run on an SDS-PAGE gel. The gel was then transferred, blotted for both NPC1 and EBOV GP, and imaged for fluorescent signal. As predicted, uncleaved GP (∼130 kDa) did not co-precipitate with NPC1 [20], [24]. (B) The intensities of the GP, GP_19 kDa_, and NPC1 bands from each sample of the blot shown in Fig. 7A were quantified and GP or GP_19 kDa_ was normalized to its respective NPC1 band signal. The experiment was conducted four times with similar results, and a representative experiment is shown.

Compound 3.47, the piperazine EBOV entry inhibitor, blocks binding of cathepsin-primed EBOV GP to NPC1 *in vitro* [20]. Curiously, it also causes cholesterol accumulation in LE/Lys ([20], and unpublished data). We therefore asked whether clomiphene, Ro 48-8071, or U18666A act similarly. In contrast to compound 3.47, however, none of the CADs tested inhibited primed GP binding to NPC1 (nor, as expected, did the cathepsin inhibitor E64d) (Fig. 7). Collectively, the results in Figs. 5, 6, and 7 suggest that the CADs that block EBOV entry do so by perturbing an NPC1-dependent pathway without, however, disrupting the direct interaction between NPC1 and primed EBOV GP.

### Three Possible Modes by which CADs Inhibit EBOV Entry

Here, we have shown that six CADs inhibit EBOV infection by blocking GP-mediated entry: AY-9944, clomiphene, Ro 48-8071, U18666A, terconazole, and triparanol (Figs. 1 and 2, Table 1). Moreover, there are at least four other CADs that inhibit EBOV infection, likely by blocking entry ([19], [49] and unpublished data). For the three CADs that we characterized in detail (clomiphene, Ro 48-8071, U18666A), we found that they all (a) block a late stage of entry (i.e., at or close to the step of membrane fusion) and (b) exert their effect through an NPC1-dependent pathway, but (c) do not impede the interaction between primed EBOV GP and NPC1. How then do these CADs inhibit EBOV entry, and in what way is NPC1 involved?

**Figure 8:**
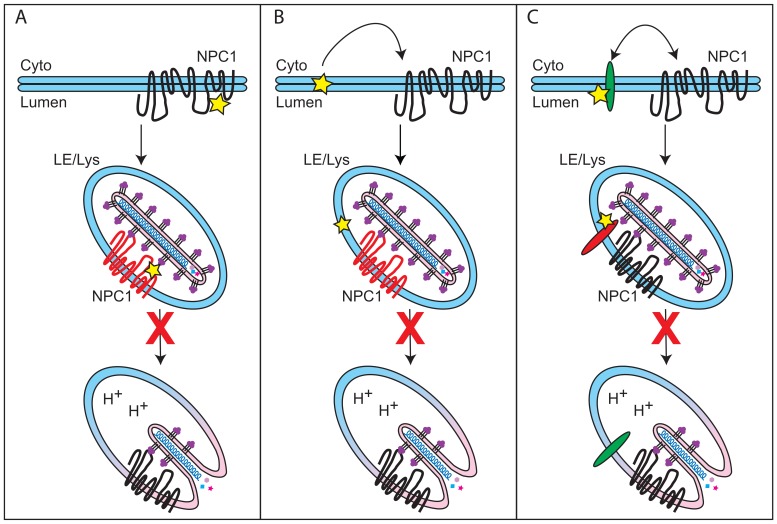
Models for how CADs May Block EBOV Entry. (A) In the first model, the CADs (yellow star) interact directly with NPC1, but at a site distinct from the C loop of NPC1 (which binds GP_19 kDa_). Binding to NPC1 inhibits a second function of NPC1 (i.e. in addition to its role in binding GP_19 kDa_) that is critical for EBOV entry. (B) In the second model, the CADs intercalate into the LE/Lys membrane, indirectly inhibiting a second function of NPC1 that promotes EBOV entry. (C) In a final model, the CADs disrupt a target distinct from NPC1 that is critical for EBOV fusion with LE/Lys) and is regulated by NPC1. The target may be another LE/Lys protein (e.g. ASM) or a lipid of the LE/Lys membrane system. (See text for details.) Alternatively, the CADs may interfere with NPC1-dependent membrane trafficking [47], [50] such that the virus is never found in an NPC1-containing compartment. In all of the models, the yellow star denotes a CAD and red in each middle image denotes the target molecule.

We envision three ways in which CADs might perturb EBOV entry in an NPC1-dependent manner (Fig. 8). In the first model (Fig. 8A), NPC1 is the direct target of the CADs, but they bind to a site distinct from the C-loop of NPC1, the binding site for primed GP [24]. Support for this possibility is provided by fluorescence spectroscopy experiments showing an interaction between U18666A and purified NPC1 [23]. According to model A, CAD binding to NPC1 compromises a second function of NPC1 in EBOV entry (i.e., in addition to binding primed GP). This function could be an NPC1-dependent modulation of the membrane or luminal composition of the LE/Lys [50]–[52] that renders the LE/Lys supportive of primed-GP mediated fusion [25]. Alternatively, the CADs may block the ability of NPC1 to induce a required conformational change in GP.

A second possible mechanism of action for the CADs (Fig. 8B) stems from their propensity to intercalate into membranes [53], [54]. Such intercalation can disrupt membrane order and affect the function of integral membrane proteins. A precedent for such a scenario has been reported for two membrane proteins, P-glycoprotein [55] and a potassium ion channel [56]. Similarly, by intercalating into the LE/Lys membrane, the CADs may indirectly affect a purported second function of NPC1 necessary for EBOV entry.

A third means by which CADs could block EBOV entry is by interacting (directly or indirectly) with a distinct (i.e., not NPC1) target (protein or membrane) in LE/Lys whose function is not only required for EBOV entry, but also is regulated by NPC1 (Fig. 8C). For example, a potential target could be acid sphingomyelinase (ASM), a positively charged enzyme that interacts with negatively charged phospholipids in LE/Lys [57]. According to several reports, in response to (positively charged) CADs, ASM dissociates from LE/Lys membranes and is then degraded by acid hydrolases in the lumen of the LE/Lys [58]–[60]. A similar set of events has been reported for another lysosomal enzyme in response to CADs [61]. Deficiencies in ASM cause cholesterol accumulation in LE/Lys and the genetic diseases Niemann Pick Types A and B [62]. Moreover, there is evidence for cross-regulation of NPC1 and ASM; it has been reported that ASM activity is reduced ∼50% in NPC1-deficient cells, and addition of exogenous ASM to NPC1-deficient cells largely rescues their defect in cholesterol egress [63]. Interestingly, a recent study has implicated ASM as a critical factor for EBOV infection [49]. In this respect, it is further interesting that while low pH, cathepsins, and NPC1 are essential for EBOV entry, collectively these three endosomal factors are not sufficient to trigger EBOV fusion ([24], [25] and White lab, unpublished data,). This suggests that at least one additional endosomal factor, perhaps ASM, might be required to support EBOV fusion.

### Summary

In this study we have presented two major findings. The first is that among eleven small molecules, there is a strict correlation between (a) their chemical structures and their abilities to: (b) induce cholesterol accumulation in LE/Lys, (c) potently (>91%) block a late stage of EBOV VLP entry, and (d) block infection by replication competent EBOV. All of the potent inhibitors are CADs with similar MW, clogP, and p*K*_a_ values, and most are class II CADs with at least one tertiary amine group (Table 1, Supp. Fig. S1). The second major result is that the CADs tested inhibit EBOV entry through an NPC1-dependent pathway, but by a mechanism that differs from the primary mode of action of compound 3.47, a piperazine that blocks binding of primed GP to NPC1 [20]. Our findings have two implications. The first is that there are at least two ways to interfere with an NPC1-dependent pathway that can block EBOV entry. Speculatively, this suggests to us that NPC1 may play more than one role in EBOV entry [25]. The second implication deals with further drug screening efforts. Collectively we and others have now identified ten CADs that inhibit EBOV, and six of these are FDA approved ([19], [49] and manuscript in preparation). As there are many other FDA-approved CADs, a data mining effort (for CADs with the chemical properties described above) may yield additional compounds to assess for potential repurposing to combat ebolavirus infections.

## Supporting Information

Figure S1 **Structures of the eleven compounds analyzed in this study.**
(TIF)

Figure S2 **Effect of CADs on NPC1 expression levels.** SNB19 cells were treated for 1 hour at 37°C with 0.1% DMSO (Mock) or 5 µM Clomiphene, Ro 48-8071, or U18666A. After harvesting the cells, LE/Lys were isolated and quantitated (by BCA assay) as described in the Materials and Methods section. Equal amounts of LE/Lys were then solubilized with 10 mM CHAPSO in TNE buffer for 20 minutes on ice, and debris was removed by centrifugation at 21,100×g for 10 min at 4°C. Samples were then denatured in SDS sample buffer with 10% BME, and 20 µg of each were run on an Any KD® SDS-PAGE gel (Biorad). Proteins were transferred to nitrocellulose, and blotted with a polyclonal antibody against NPC1 (ThermoFischer Scientific: PA1-16817). Protein blots were imaged on an Odyssey® infrared imaging system (LI-COR Biosciences), and NPC1 band intensities were quantified using Odyssey application software (version 3.0.16) and reported as the band intensity normalized to that of mock-treated samples. Values are the averages from two replicate gel samples for experiment 1 and from a single gel sample for experiment 2. A representative blot (from experiment 1) is shown above the NPC1 band intensity values.
(TIF)
