# Correction: Multiple Cationic Amphiphiles Induce a Niemann-Pick C Phenotype and Inhibit Ebola Virus Entry and Infection

**DOI:** 10.1371/annotation/76780c06-ac81-48a3-8cce-509da6858fe5

**Published:** 2013-10-01

**Authors:** Charles J. Shoemaker, Kathryn L. Schornberg, Sue E. Delos, Corinne Scully, Hassan Pajouhesh, Gene G. Olinger, Lisa M. Johansen, Judith M. White

There is an error in the legend for Figure 6, "CADs inhibit EBOV GP-mediated infection in an NPC1-dependent manner." The complete, correct Figure 6 legend is:Parental CHO cells (─────) and stably overexpressing CHO NPC1 cells (− − −) were pre-treated with the indicated concentration of inhibitor for 1 hr at 37°C, and then infected with VSV-GPΔ for 18 hr in the continued presence of inhibitor. Each concentration of inhibitor was tested (in duplicate) in the following number of experiments: E64d (n = 2), compound 3.47 (n = 2), clomiphene (n = 3), Ro 48-8071 (n = 3), and U18666A (n = 4). Infection values were normalized to DMSO treated samples and averaged across experiments. Error bars represent standard error. 

