# Supplementary figures and images for: Multiple Cationic Amphiphiles Induce a Niemann-Pick C Phenotype and Inhibit Ebola Virus Entry and Infection
Source: PLoS One. 2013 Feb 18;8(2):e56265. doi: 10.1371/journal.pone.0056265 (PMC3575416; doi:10.1371/journal.pone.0056265)

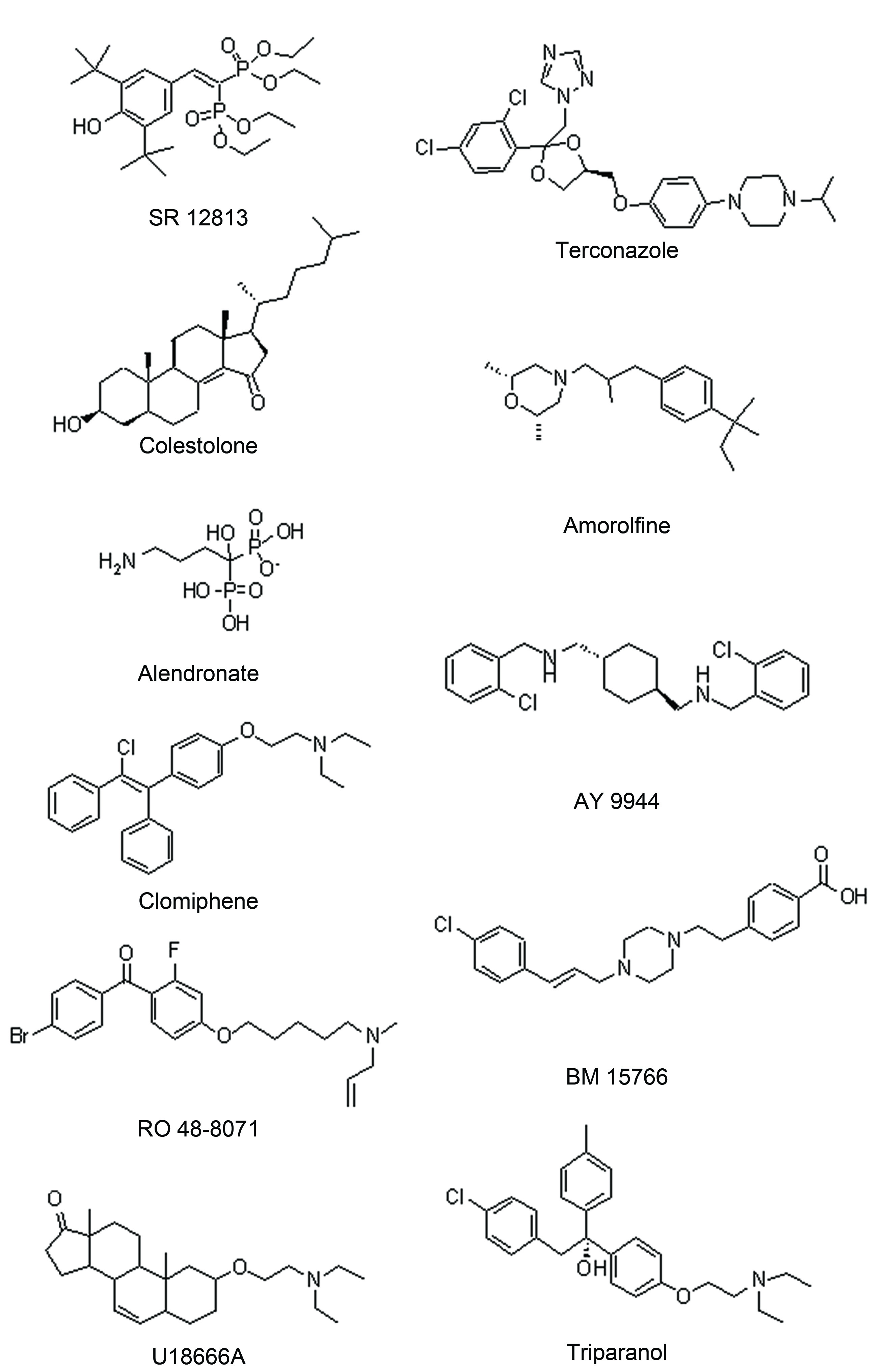

Supplement: Figure S1 — Structures of the eleven compounds analyzed in this study. (TIF) [file pone.0056265.s001.tif]

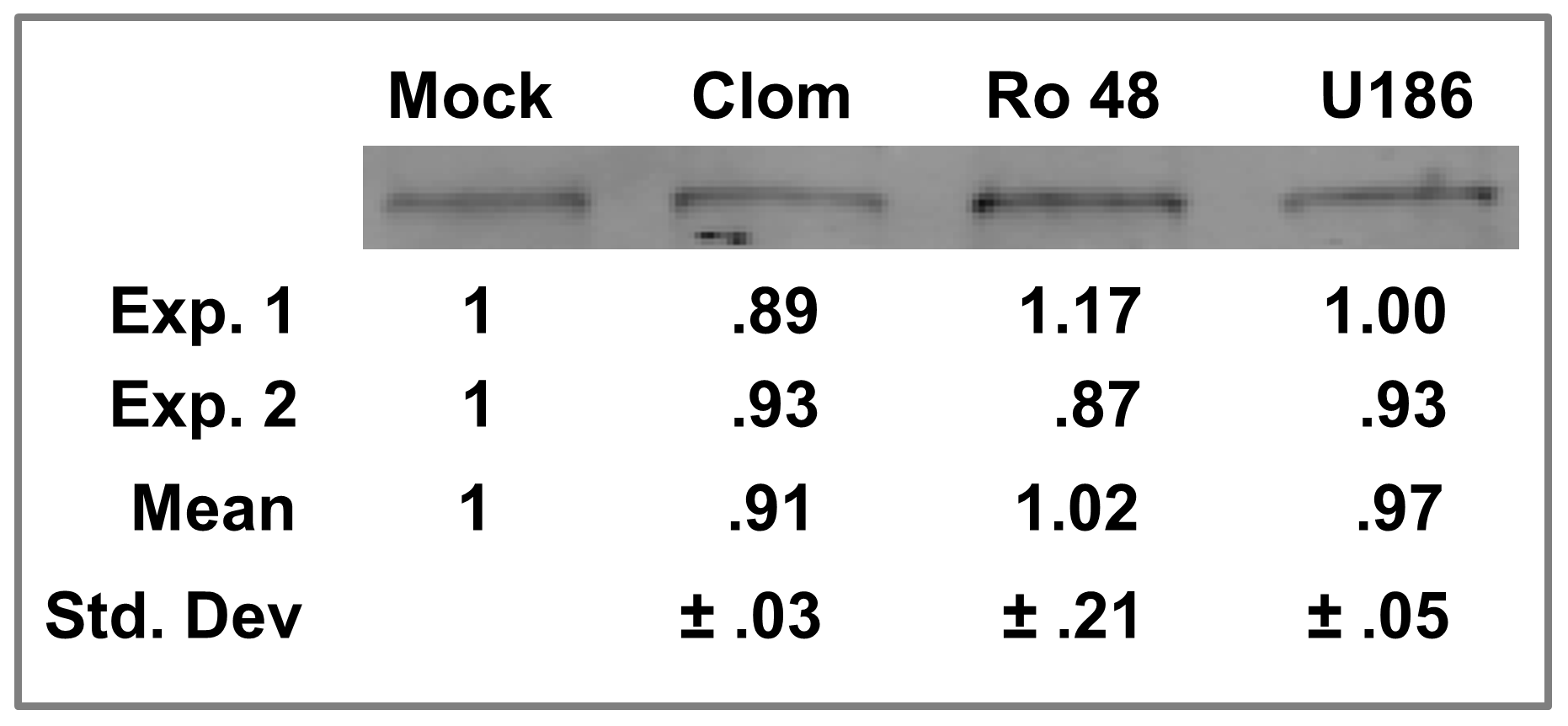

Supplement: Figure S2 — Effect of CADs on NPC1 expression levels. SNB19 cells were treated for 1 hour at 37°C with 0.1% DMSO (Mock) or 5 µM Clomiphene, Ro 48-8071, or U18666A. After harvesting the cells, LE/Lys were isolated and quantitated (by BCA assay) as described in the Materials and Methods section. Equal amounts of LE/Lys were then solubilized with 10 mM CHAPSO in TNE buffer for 20 minutes on ice, and debris was removed by centrifugation at 21,100×g for 10 min at 4°C. Samples were then denatured in SDS sample buffer with 10% BME, and 20 µg of each were run on an Any KD® SDS-PAGE gel (Biorad). Proteins were transferred to nitrocellulose, and blotted with a polyclonal antibody against NPC1 (ThermoFischer Scientific: PA1-16817). Protein blots were imaged on an Odyssey® infrared imaging system (LI-COR Biosciences), and NPC1 band intensities were quantified using Odyssey application software (version 3.0.16) and reported as the band intensity normalized to that of mock-treated samples. Values are the averages from two replicate gel samples for experiment 1 and from a single gel sample for experiment 2. A representative blot (from experiment 1) is shown above the NPC1 band intensity values. (TIF) [file pone.0056265.s002.tif]
